# Nbs1‐mediated DNA damage repair pathway regulates haematopoietic stem cell development and embryonic haematopoiesis

**DOI:** 10.1111/cpr.12972

**Published:** 2021-02-14

**Authors:** Yu Chen, Jie Sun, Zhenyu Ju, Zhao‐Qi Wang, Tangliang Li

**Affiliations:** ^1^ Institute of Aging Research School of Medicine Hangzhou Normal University Hangzhou China; ^2^ Jiangsu Hansoh Pharmaceutical Group Co., Ltd. Lianyungang China; ^3^ Leibniz Institute on Aging ‐ Fritz Lipmann Institute Jena Germany; ^4^ Faculty of Biology and Pharmacy Friedrich‐Schiller University of Jena Jena Germany; ^5^ State Key Laboratory of Microbial Technology Shandong University Qingdao China; ^6^ NHC Key Laboratory of Birth Defect for Research and Prevention Hunan Provincial Maternal and Child Health Care Hospital Changsha China

**Keywords:** Cell fate, DNA damage response, Haematopoietic stem cells, Nbs1, p53

## Abstract

**Objectives:**

DNA damages pose threats to haematopoietic stem cells (HSC) maintenance and haematopoietic system homeostasis. Quiescent HSCs in adult mouse bone marrow are resistant to DNA damage, while human umbilical cord blood‐derived proliferative HSCs are prone to cell death upon ionizing radiation. Murine embryonic HSCs proliferate in foetal livers and divide symmetrically to generate HSC pool. How murine embryonic HSCs respond to DNA damages is not well‐defined.

**Materials and methods:**

Mice models with DNA repair molecule Nbs1 or Nbs1/p53 specifically deleted in embryonic HSCs were generated. FACS analysis, in vitro and in vivo HSC differentiation assays, qPCR, immunofluorescence and Western blotting were used to delineate roles of Nbs1‐p53 signaling in HSCs and haematopoietic progenitors.

**Results:**

Nbs1 deficiency results in persistent DNA breaks in embryonic HSCs, compromises embryonic HSC development and finally results in mouse perinatal lethality. The persistent DNA breaks in Nbs1 deficient embryonic HSCs render cell cycle arrest, while driving a higher rate of cell death in haematopoietic progenitors. Although Nbs1 deficiency promotes Atm‐Chk2‐p53 axis activation in HSCs and their progenies, ablation of p53 in Nbs1 deficient HSCs accelerates embryonic lethality.

**Conclusions:**

Our study discloses that DNA double‐strand repair molecule Nbs1 is essential in embryonic HSC development and haematopoiesis. Persistent DNA damages result in distinct cell fate in HSCs and haematopoietic progenitors. Nbs1 null HSCs tend to be maintained through cell cycle arrest, while Nbs1 null haematopoietic progenitors commit cell death. The discrepancies are mediated possibly by different magnitude of p53 signaling.

## Graphical abstract

Genetic deletion of Nbs1, a DNA double‐strand break repair molecule, in murine embryonic haematopoietic stem cells (HSCs) generates persistent DNA damages in HSCs and their progenies, contributing to defective haematopoiesis and perinatal lethality in mice. Nbs1 null HSCs and haematopoietic progenitors show different cell fate, which could be mediated by differential magnitude of p53 signaling in each cell population. Intriguingly, p53 loss accelerates lethality of mice with Nbs1 deletion in HSCs, indicating an essential role of p53 signaling safeguarding embryonic haematopoiesis.

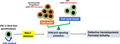

## 1 INTRODUCTION

Cells from adult animal tissues are constantly threatened by the DNA damages generated endogenously by DNA replication, ROS production via mitochondria, etc, and exogenously through exposure to DNA damage inducing chemicals and environmental radiation.^1^, ^2^ Multiple mechanisms have been evolved to repair different types of DNA damages.^2^, ^3^, ^4^, ^5^ Deficiencies in components of DNA damage response and repair pathways result in accumulations of genomic abnormality, compromised cellular fitness, and even tumorigenesis.^1^ Current studies indicate that DNA damage response functions beyond the repair of DNA lesions, but rather generates systematic effects, including epigenetic modulation, transcription regulation and cell cycle regulation.^6^

Stem cells, residing in different tissues, could self‐renew to retain the stem cell identity and differentiate into certain somatic lineages in order to maintain tissue functions.^7^ Studies from mouse models with DNA repair deficiencies indicate that genomic stability maintenance is essential for the stem cell function and tissue homeostasis.^2^, ^8^ haematopoietic stem cell (HSC), as a prototype in stem cell biology research, guards the haematopoietic system homeostasis and prevents the premature ageing by sustaining self‐renewal and differentiation capabilities.^9^, ^10^, ^11^, ^12^ During the mammalian development, definitive HSCs emerge from the aorta‐gonads‐mesonephros (AGM) and further migrate to foetal livers. HSCs in foetal livers (namely foetal liver HSCs, FL‐HSCs) are cycling and divide to establish the HSC pool for the adulthood.^13^, ^14^ In adult life, HSCs are mostly quiescent and localized in the bone marrow niches composing of mesenchymal stromal cells and endothelial cells.^13^, ^15^ In mammals, accumulation of DNA double‐strand breaks (DSBs) has been found in HSCs from aged mice and humans.^16^, ^17^ Since HSCs in aged animals show abnormal self‐renewal activity and poor haematopoietic system reconstitution ability, DNA damage has been implicated as one of the limiting factors in functional maintenance of HSCs.^8^, ^11^, ^16^, ^18^ In line with this hypothesis, deficiencies in DNA repair molecules, such as *Atm*,^19^ *Rad50*,^20^ *Mre11*,^21^ *Fancd2*,^22^, ^23^ *Brca1* ^24^ *and Xpg*,^25^ generate high frequency of DNA lesions in HSCs, resulting in progressive loss of HSCs and compromised haematopoiesis. It is interesting to note that HSCs and their derived haematopoietic progenitors have distinct response to DNA damages. For example, Mohrin *et al* found that murine quiescent HSCs are resistant to DNA damage‐induced cell death. However, proliferating haematopoietic progenitors are hypersensitive to DNA damages and prone to commit cell death.^26^ Gene expression analysis on purified HSCs and proliferating haematopoietic progenitors shows that HSCs express higher level of pro‐survival genes and lower level of pro‐apoptotic genes.^26^ Intriguingly, proliferative HSCs isolated from human umbilical cord blood samples (UCB‐HSCs) are hypersensitive to low dose of IR as compared to UCB‐HSC derived haematopoietic progenitors,^27^ suggesting that proliferative human UCB‐HSCs with the embryonic origin may have distinct regulatory mechanism in cell fate determination upon DNA damages.^28^, ^29^ It is proposed that distinct cellular response to DNA damages in human and mouse HSCs could be either species‐specific or cell cycle specific, since DNA repair efficiency varies between species and could be affected by the cell cycle status.^30^, ^31^ Thus, direct analysis on DNA damage response in proliferative HSCs from mouse foetus is necessary to solve these debates.

In murine foetal liver, active cycling HSCs generate high level of replicative stress. Thus, foetal liver HSCs may require more proficient DDR machinery to safeguard HSC expansion and proper haematopoiesis. Whether and how DDR could affect foetal haematopoiesis are poorly understood. We are motivated to investigate DDR and its biological consequences in cycling embryonic HSCs in foetal livers. DNA double‐strand break is the most dangerous type of DNA damage to a cell.^32^, ^33^ Inaccurate DSB repair results in chromosome translocations, and failure to repair DSBs could cause cell death. NBS1, mutated in Nijmegen Breakage Syndrome,^34^ a human autosomal recessive genetic disorder, plays key roles in DSB signaling and repair.^35^ Mechanistically, NBS1, together with MRE11 and RAD50 (MRN), senses DNA double‐strand breaks and transduces the DNA damage signals for cell cycle arrest and the repair of DNA lesions.^35^ Furthermore, MRN functions in DNA replication by resolving DNA replication intermediate, failure of which induces p53‐dependent cell cycle arrest and cell death.^36^ NBS patients show symptoms of growth retardation, microcephaly and immunodeficiency, which could be recapitulated by mouse models with Nbs1 specific deletion in central nervous system, T‐ and B‐cell progenitors.^34^, ^37^, ^38^, ^39^ Mutation in NBS1 is associated with paediatric aplastic anaemia,^40^ suggesting a role of NBS1 in foetal and perinatal development of the haematopoietic system. However, the Nbs1‐mediated DDR in foetal haematopoiesis remains unknown. In this study, we investigated biological consequences of persistent DSBs induced by Nbs1 deletion in FL‐HSCs and haematopoietic progenitors and defined Nbs1 functions in embryonic haematopoiesis. We found that the strength of p53 signaling activation upon Nbs1 deficiency dictates differential fates of embryonic HSCs and haematopoietic progenitors during embryonic development.

## 2 MATERIALS AND METHODS

### 2.1 Mice and genotyping strategies

By crossing the *Nbs1* ^f/f^ mice with Vav‐Cre transgenic mice,^37^, ^41^ we generated *Nbs1* ^f/f^ Vav‐Cre^+^ mice, designated as Nbs1‐HSCΔ. Since mice with at least one intact Nbs1 allele (*Nbs1* ^f/+^ Vav‐Cre^+^) did not show any defect in embryonic haematopoiesis (Figure S1A‐D), mice with genotypes of *Nbs1* ^+/+^ Vav‐Cre^+^, *Nbs1* ^+/+^ Vav‐Cre^‐^, *Nbs1* ^f/+^ Vav‐Cre^+^, *Nbs1* ^f/+^ Vav‐Cre^‐^ and *Nbs1* ^f/f^ Vav‐Cre^‐^ are grouped as the controls (Co). Additionally, *Nbs1* ^f/+^ Vav‐Cre^+^ mice were crossed with *p53* knockout mice to generate Nbs1‐HSCΔ; *p53^‐/‐^* mice (Nbs1/p53‐HSCΔ).^39^ All animals were maintained under specific pathogen‐free conditions. Animal care and experiments were performed in accordance with the ethics committee guideline. For the *Nbs1* loci genotyping, three primers were used to identify wild‐type allele, *Nbs1* ^F^ allele (0.3kb) and *Nbs1* deleted (∆) allele (0.6kb)^39^: loxPtestR, 5′‐AATACAGTGACTCCTGGAGG‐3′; Intron5F, 5′‐ATAAGACAGTCACCACTGCG‐3′; Exon6, 5′‐CAGGGCGACATGAAAGAAAAC‐3′. p53 knockout allele was detected by PCR using the following primers^39^: X7, 5′‐TATACTCAGAGCCGGCCT‐3′; X6.5, 5′‐ACAGCGTGGTGGTACCTTAT‐3′; Neo, 5′‐CATTCAGGACATAGCGTTGG‐3′. The Vav‐cre transgene genotyping was conducted with the primer set: vav‐1, 5′‐GCCTGCCCTCCCTGTGGATGCCACCT‐3′; vav‐2, 5′‐GTGGCAGAAGGGGCAGCCACACCATT‐3′.

### 2.2 Characterization of HSCs and haematopoietic progenitors from mouse embryonic foetal livers and bone marrows from neonatal mice

foetal livers at different developmental stage were isolated, minced, and passed three times through 26‐gauge needles to release foetal liver haematopoietic cells. The enucleated red blood cells were removed by 1 × ACK buffer as described.^39^ Cell surface marker combinations used for FACS analysis and sorting are: foetal liver HSC, Lineage ^‐^ (CD4, CD8, B220, Gr1, Ter119), Sca1^+^, c‐Kit^+^, CD11b^+^; foetal liver haematopoietic progenitors (LK progenitors), Lin^‐^ (CD4, CD8, B220, Gr1, Ter119), Sca1^‐^, c‐Kit^+^. Cell death index was characterized by staining cell population with Annexin‐V antibody (BD Biosciences), and cell cycle status was determined by staining the sorted HSCs and haematopoietic progenitors with Ki67 antibody (NeoLabs).

For the FACS analysis in the neonatal mice, femur and tibia bones were isolated and grinded in FACS buffer (1 × PBS with 2% FBS). Cell surface marker combinations used for FACS analysis on neonatal mice are: HSC, Lineage^‐^ (CD4, CD8, B220, Gr1, Ter119, CD11b), Sca1^+^, c‐Kit^+^; haematopoietic progenitors (LK progenitors), Lineage^‐^ (CD4, CD8, B220, Gr1, Ter119, CD11b), Sca1^‐^, c‐Kit^+^.

### 2.3 Chromosome analysis

Chromosome analysis was conducted by karyotyping chromosome metaphases from E15.5 foetal liver haematopoietic cells. Briefly, pregnant mice with E15.5 mouse embryos were intraperitoneally injected with colcemid (Sigma‐Aldrich, 100ug per mouse). After 3 hours, foetal livers were isolated, minced and passed 3 times with 26‐gauge needles to make single cell suspension. The metaphase preparation followed the protocol as previously described.^39^, ^42^ For the chromosome analysis, metaphases were stained with Giemsa solution (5% Giemsa in 0.025M KH_2_PO4, pH7.0) and images of metaphases were captured with a Zeiss Axio Imager M1 microscope and analysed by Bandview software (Applied Spectral Imaging System).

### 2.4 Western blotting and immunofluorescence analysis

Western blotting analysis was conducted following the procedure as previously described.^39^ Briefly, foetal liver samples at indicated developmental stages were homogenized in RIPA buffer (Sigma‐Aldrich) supplemented with Protease/Phosphatase inhibitors (APExBIO). Protein samples (40‐80ug) were separated with 10%‐15% of SDS‐PAGE gel. The following primary antibodies are used in this study: rabbit anti‐Nbs1 (1:1000, Cell Signaling; or a homemade Nbs1 antibody); rabbit anti‐p53 (Ser18) (1:1000, Cell Signaling); mouse anti‐γ‐H2AX (1:5000, Merck Millipore); mouse anti‐Chk2 (1:1500, Merck Millipore); mouse anti‐β‐Actin (1:5000, Proteintech). The secondary antibodies used are goat anti‐rabbit HRP and goat anti‐mouse HRP (1:2500, Proteintech).

For immunofluorescence analysis on DNA double‐strand breaks (DSBs) in HSCs and haematopoietic progenitors (LK progenitors), cells were FACS sorted onto gelatin‐coated coverslips. The subsequent fixation and antibody staining procedure followed the published protocol.^43^ The mouse anti‐γ‐H2AX antibody (1:800, Merck Millipore) was used to investigate DSBs in each cell population.

### 2.5 Histological analysis

foetal livers at indicated developmental stages, and tibia/femur bone samples from control and Nbs1‐HSCΔ mice were fixed overnight with 4% paraformaldehyde (PFA) (pH 7.2) at 4 degree and then processed into paraffin sections. Sections of 5um thickness were stained with haematoxylin and eosin (H&E). Images were taken with a Zeiss M1 microscope and processed with Zeiss Zen software (Zeiss, Jena, Germany).

### 2.6 Foetal liver HSC transplantation assay

1 × 10^6^ unfractionated foetal liver haematopoietic cells, including HSCs and haematopoietic progenitors, from E14.5 control or Nbs1‐HSC∆ mice embryos (Ly5.2 background) were intravenously injected into lethally irradiated wild‐type recipient mice (Ly5.1 background). The reconstituted mice were monitored for mortality. Mice were sacrificed at indicated time point to investigate the reconstitution efficiency by FACS analysis on expressions of Ly5.1 and Ly5.2.

### 2.7 In vitro haematopoietic colony formation assay

E14.5 foetal liver cells were used for in vitro colony formation assay in the semi‐solid methylcellulose medium (MethoCult^TM^ M3434, STEMCELL Technologies). Colonies of BFU‐E (Burst‐forming unit‐erythroid), CFU‐GM (Colony‐forming unit‐granulocyte, macrophage) and CFU‐GEMM (Colony‐forming unit‐granulocyte, erythroid, macrophage and megakaryocyte) were scored according to the company manual.

### 2.8 qRT‐PCR

The quantitative RT‐PCR (qRT‐PCR) was conducted according to a published protocol.^44^ Briefly, RNAs were isolated from FACS‐purified HSCs and haematopoietic progenitors (LK progenitors) with Total RNA Miniprep Kit (Sigma‐Aldrich) and cDNAs were synthesized using the SuperScript® III Reverse Transcriptase (Invitrogen) according to the company manual. qRT‐PCR in triplicate for each sample was performed using Platinum SYBR Green qPCR SuperMix‐UDG (Invitrogen) on the LightCycle®480 Real‐Time PCR system (Roche). The primers used for PCR amplifications are listed below. The expression of β‐Actin was used as the internal control.

p21: Fwd, 5′‐GCAGATCCACAGCGATATCC‐3′; Rev, 5′‐CAACTGCTCACTGTCCACGG‐3′;

p53: Fwd, 5′‐TGGAAGACTCCAGTGGGAA‐3′; Rev, 5′‐TCTTCTGTACGGCGGTCTCT‐3′;

Noxa: Fwd, 5′‐GGAGTGCACCGGACATAACT‐3′; Rev, 5′‐TTGAGCACACTCGTCCTTCA‐3′;

Bax: Fwd, 5′‐TGGAGCTGCAGAGGATGATTG‐3′; Rev, 5′‐AGCTGCCACCCGGAAGA‐3′;

β‐Actin: Fwd, 5’‐AGAGGGAAATCGTGCGTGAC‐3’; Rev, 5’‐CAATAGTGATGACCTGGCCGT‐3’.

## 3 RESULTS

### 3.1 Generation a mouse model with Nbs1 specifically deleted in developing HSCs and their progenies

To study the role of DNA repair molecule Nbs1 in embryonic HSCs and haematopoiesis, we crossed *Nbs1^f/f^* mice with Vav‐Cre transgenic mice. Since Cre transgene expression driven by Vav promoter starts in foetus and foetal liver is the major organ for embryonic haematopoiesis, we thus collected mouse livers at different embryonic stages for our analysis. To confirm the deletion of Nbs1, we conducted PCR analysis of the Nbs1 alleles with foetal liver cells and FACS‐purified c‐Kit^+^ CD34^+^ cells (including foetal liver HSCs and haematopoietic progenitors),^45^ and Western blot analysis of Nbs1 protein in E14.5‐15.5 foetal livers from *Nbs1^f/+^*Vav‐Cre^‐^, *Nbs1^f/+^*Vav‐Cre^+^ and *Nbs1^f/f^*Vav‐Cre^+^ mice. We found that HSC and its progenies from *Nbs1^f/f^*Vav‐Cre^+^ mouse embryonic foetal livers (Figure S1A‐B, E‐F) had efficient deletion of the *Nbs1* gene. Of note, *Nbs1^f/+^*Vav‐Cre^+^ foetal liver cells showed efficient deletion of one Nbs1 locus and reduction of half amount of Nbs1 protein (Figure S1A‐B). However, loss of one Nbs1 locus did not affect embryonic haematopoiesis as indicated by normal foetal liver cellularity and mature haematopoietic cell productions in *Nbs1^f/+^Vav‐Cre^+^* foetus (Figure S1C‐D). Thus, we designated *Nbs1^f/f^*Vav‐Cre^+^ mice as Nbs1‐HSCΔ, while the control group in our analysis includes mice with genotypes of *Nbs1^+/+^*Vav‐Cre^‐^, *Nbs1^+/+^*Vav‐Cre^+^, *Nbs1^f/+^*Vav‐Cre^‐^, *Nbs1^f/+^*Vav‐Cre^+^ and *Nbs1^f/f^*Vav‐Cre^‐^.

**Figure 1:**
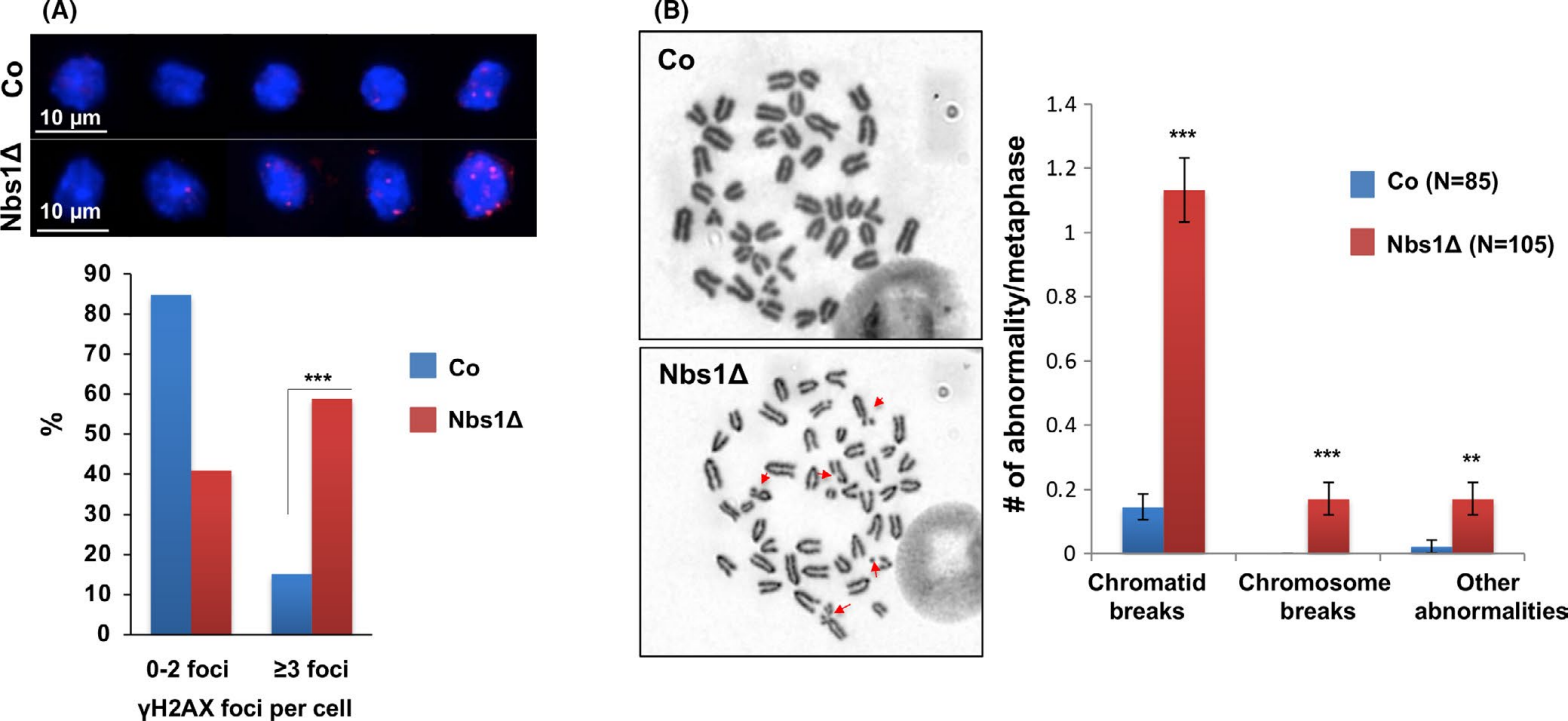
Nbs1 deletion generates persistent DNA breaks in HSCs. A, Genomic instability, indicated by γ‐H2AX foci (**Red**), of freshly sorted HSCs from control and Nbs1‐HSCΔ E16.5 embryos. The frequency of cells with different amount of DNA damages is summarized on the lower panel. Data are generated from HSCs isolated from 2 controls and 2 Nbs1‐HSCΔ embryos. 189 control LSK HSCs and 246 Nbs1 deficient HSCs are used for the final quantification. ***, *P* < .001. Chi‐square test is used. B, Chromosome abnormalities in E15.5 foetal liver haematopoietic cells from 3 control and 4 Nbs1‐HSCΔ embryos. Red arrows mark the chromosome abnormalities in Nbs1‐HSCΔ haematopoietic cells. ‘N’ denotes the numbers of metaphases used for the quantification (right panel). Please note: ‘Other chromosome abnormalities’ include chromosome fusions and endo‐replication

Since Nbs1 is involved in the DNA double‐strand break (DSB) repair pathway,^3^ we freshly isolated HSC and its progenies (LK progenitors) from E16.5 foetal livers and found Nbs1‐HSCΔ HSCs and LK progenitors contained higher levels of γH2AX foci, a marker of DNA double‐strand break (Figure 1A). We and other groups previously showed that Nbs1 deletion generates chromosome instability in B and T cells.^38^, ^39^ We analysed the genomic stability of Nbs1 deficient haematopoietic cells from E15.5 foetal livers and found a significant increase of chromosome abnormalities in Nbs1 null haematopoietic cells (Figure 1B), consistent with the role of Nbs1 in the maintenance of chromosome stability.^37^, ^39^ Thus, our Nbs1‐HSCΔ mouse not only serves as a specific model to study biological functions of DSB repair mechanism in foetal haematopoiesis, but also is useful for elucidating biological consequences of persistent DNA breaks during foetal HSC expansion and development.

### 3.2 Nbs1 deficiency renders defective haematopoiesis in neonatal mice

**Figure 2:**
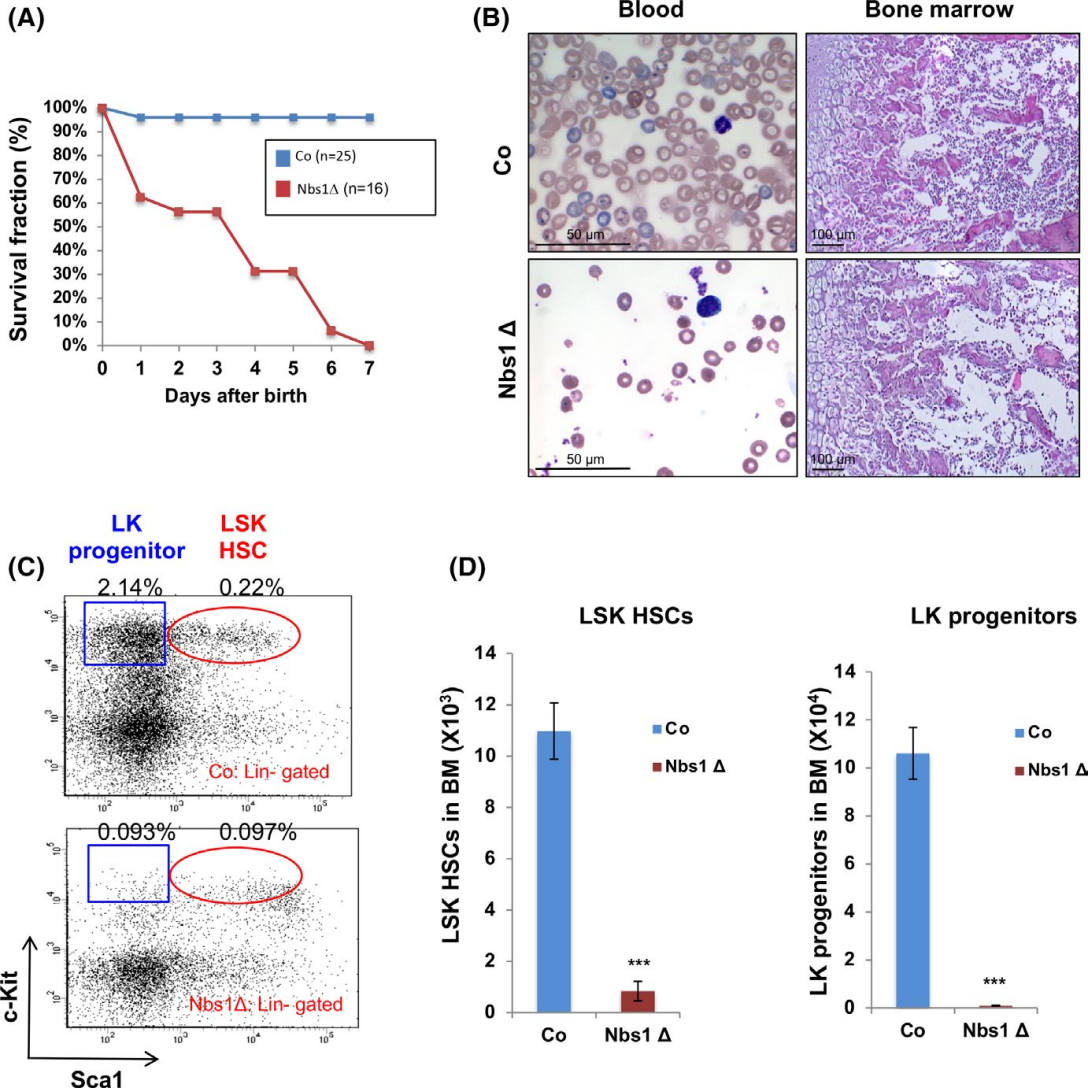
Nbs1 deletion causes perinatal lethality and depletion of HSCs in mice. A, Kaplan‐Meier survival curve of control (Co) and Nbs1‐HSCΔ (Nbs1Δ) newborns. ‘N’ denotes the number of mice used for the quantification. B, Blood smear (left panels) from P1 control (Co) and Nbs1‐HSCΔ (Nbs1Δ) mice and HE staining of tibia (right panels) from P3 control (Co) and Nbs1‐HSCΔ (Nbs1Δ) mice. C, Representative FACS profiles of haematopoietic stem cells (LSK HSCs) and haematopoietic progenitors (LK progenitors) from control and Nbs1‐HSCΔ mice. Frequencies of LK progenitors and LSK HSCs (in gated bone marrow mononuclear cells) are shown. D, Nbs1 deficiency results in depletion of HSCs (left panel, LSK HSCs) and haematopoietic progenitors (right panel, LK progenitors) in p1 control (Co) and Nbs1‐HSCΔ (Nbs1Δ) mice. Note: N = 3 for each group; ‘P1’ denotes postnatal day 1. Note: *, *P* < .05; **, *P* < .01; ***, *P* < .001. Unpaired Student's *t* test is used

Nbs1 is essential for the viability of cells and mice. The Nbs1 deletion in foetal liver HSCs severely compromised the viability of *Nbs1*‐HSCΔ embryos since only 50% of mutants could survive to birth (Figure S1G). The survived mutant mice are anaemic (Figure S2A), growth‐retarded (Figure S2B) and die quickly within 7 days after birth (Figure 2A). Macroscopic analysis showed that bone marrows from Nbs1‐HSCΔ newborns are hypocellular and aplastic (Figure 2B; Figure S2C). FACS analysis revealed around 10‐fold reductions in the number of HSCs and almost complete ablation of haematopoietic progenitors (Lin^‐^Sca1^‐^c‐Kit^+^, LK progenitors) in p1 (postnatal day 1) Nbs1‐HSCΔ mice (Figure 2C‐D). The blood smear analysis further revealed that Nbs1 deficiency compromises haematopoiesis since peripheral blood from Nbs1‐HSCΔ mice had very few numbers of mature haematopoietic cells, including erythrocytes and monocytes (Figure 2B). These data strongly indicate that Nbs1, or MRN, is essential for the haematopoiesis in neonatal mice.

### 3.3 Compromised haematopoiesis in Nbs1‐HSCΔ foetal livers

**Figure 3:**
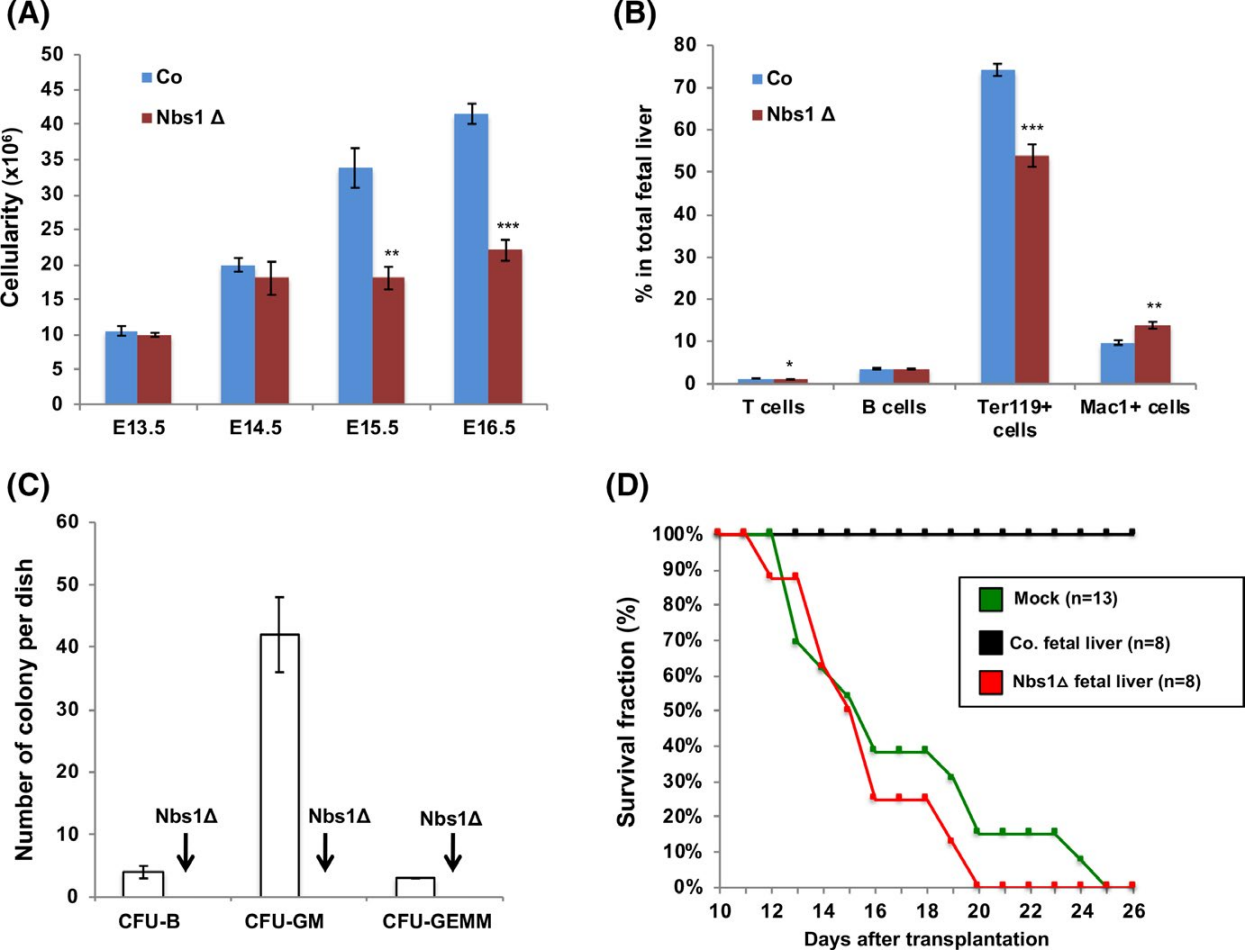
Defective foetal liver haematopoiesis after Nbs1 deletion. A, Cellularity of foetal livers from control and Nbs1‐HSCΔ embryos at different developmental stages (n ≥ 3 for each group). B, Frequencies of T cells (CD3^+^), B cell (B220^+^), red blood cells (Ter119^+^), granulocytes and macrophages (Mac1^+^) in E15.5 control (Co) and Nbs1‐HSCΔ (Nbs1Δ) foetal livers (N = 3 for each genotype). C, Colony formation assay of control and Nbs1‐HSCΔ foetal liver haematopoietic cells. Please note that Nbs1‐HSCΔ foetal liver haematopoietic cells fail to form any haematopoietic colony in vitro. D, Kaplan‐Meier survival curve of lethally irradiated Ly5.1 mice transplanted with control or Nbs1‐HSCΔ foetal liver cells. Mock reconstitution (+PBS) is used to determine the lethal dose. Please note that Nbs1‐HSCΔ foetal liver haematopoietic cells could not reconstitute the haematopoietic system in lethally irradiated Ly5.1 mice. Note: *, *P* < .05; **, *P* < .01; ***, *P* < .001. Unpaired Student's *t* test is used

Since Vav promoter drives Cre recombinase expression around E10.5 and foetal liver is the tissue where embryonic HSCs expand and embryonic haematopoiesis happens, we next analysed embryonic haematopoiesis. We found that there are constantly lower numbers of cells in Nbs1‐HSCΔ foetal livers at embryonic stages starting from E13.5 (Figure 3A). FACS analysis on E15.5 Nbs1‐HSCΔ foetal livers showed great reductions on frequencies of T cells and Ter119^+^ erythroid cells, while an over‐representation of Mac1^+^ myeloid cells was noticed (Figure 3B). These data indicate that Nbs1 deletion in foetal liver HSCs results in defective haematopoiesis.

To further investigate Nbs1’s roles in HSC self‐renewal and differentiation, we conducted in vitro haematopoietic colony formation assay and in vivo haematopoietic system reconstitution assay. Nbs1 deficient HSCs failed to differentiate into any haematopoietic cell in vitro and cannot reconstitute haematopoietic system in lethally irradiated congenic mice (Figure 3C‐D). Thus, loss of Nbs1 severely impairs HSC functionality and compromises the haematopoietic system establishment in a cell autonomous manner. These data indicate that Nbs1, or MRN, is essential for embryonic HSC self‐renewal and differentiation. Our finding strongly suggests that the proficient DDR is essential for the maintenance of embryonic HSCs and haematopoietic system homeostasis.^21^, ^46^

### 3.4 Distinct cellular fate of HSCs and haematopoietic progenitors lacking Nbs1

**Figure 4:**
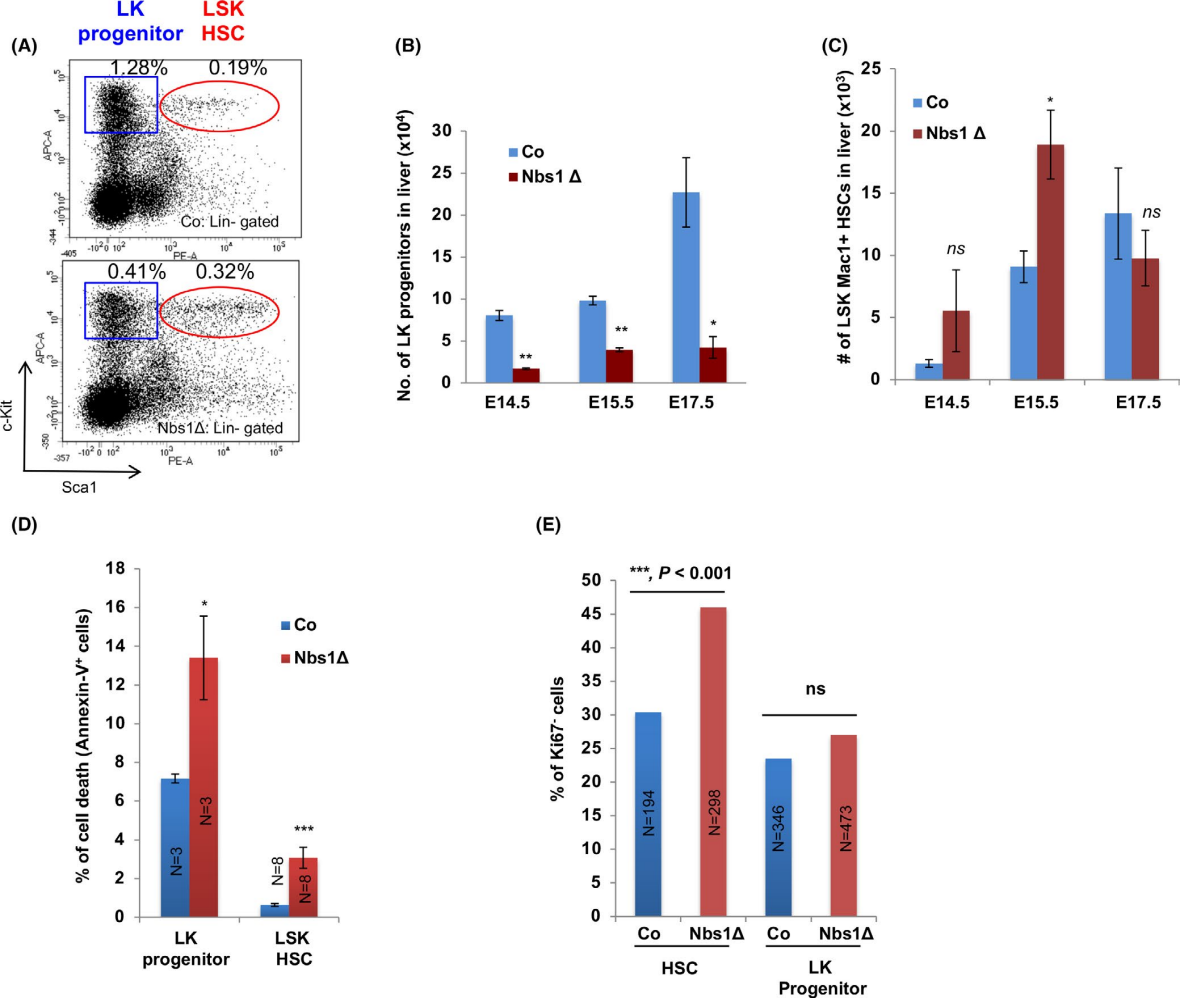
Cell fates of foetal liver HSCs and LK progenitors upon Nbs1 deletion. A, Representative FACS profile of HSCs and LK progenitors from E15.5 embryonic foetal livers of control (Co) and Nbs1‐HSCΔ (Nbs1Δ) mouse embryos. Frequencies of LK progenitors and LSK HSCs (in gated foetal liver cells) are shown. B, Absolute number of LK progenitors in foetal livers at different stage (N ≥ 3 for each group). C, Absolute number of HSCs in foetal livers at different stage (N ≥ 3 for each group). D, Apoptosis (Annexin V^+^) of LK progenitors and HSCs from E15.5 control and Nbs1‐HSCΔ foetal livers. ‘N’ denotes the number of embryos analysed. E, Ki67^‐^ cells in HSCs and LK progenitors at E16.5. HSCs and LK progenitors sorted from 2 controls and 2 mutant embryos are used for the analysis. N denotes the number of cells used for the quantification (chi‐square test is used. ***, *P* < .001; ns, not significant). Note: *, *P* < .05; **, *P* < .01; ***, *P* < .001. Unpaired Student's *t* test is used

Previous studies showed that mutations of DSB repair genes, such as *Atm* and *Rad50*, cause progressive loss of HSCs and their progenies.^16^, ^21^, ^47^ In newborn Nbs1‐HSCΔ mice, HSCs and LK progenitors were all dramatically reduced, representing around 8% and 0.75% as those in controls (Figure 2C‐D). Intriguingly, although LK progenitors in Nbs1‐HSCΔ foetal livers were constantly less than controls (Figure 4A‐B), a short time window around E15.5 with transient increase of HSC numbers was observed (Figure 4A, 4C), suggesting that embryonic HSCs and LK progenitors have distinct response to the DNA damages. Annexin‐V antibody staining on freshly isolated haematopoietic cell populations from Nbs1‐HSCΔ foetal livers revealed a significantly higher magnitude of cell death in the Nbs1 deficient LK progenitors, while only a mild increase of apoptotic index was found in HSCs (Figure 4D). This observation indicates that cycling foetal liver HSCs, similar to quiescent HSCs during adulthood, are resistant to DNA damage‐induced cell death as compared with LK progenitors.^26^ In accordance to this finding, Ki67 antibody staining on sorted HSCs and LK progenitors from control and Nbs1‐HSCΔ foetal livers showed that Nbs1 deficiency generated higher frequency of cell cycle arrested HSCs (Ki67^‐^ HSCs), while resulting in no change on the cell cycle status of LK progenitors (Figure 4E).

### 3.5 P53 null accelerates the lethality of Nbs1‐HSCΔ mice

**Figure 5:**
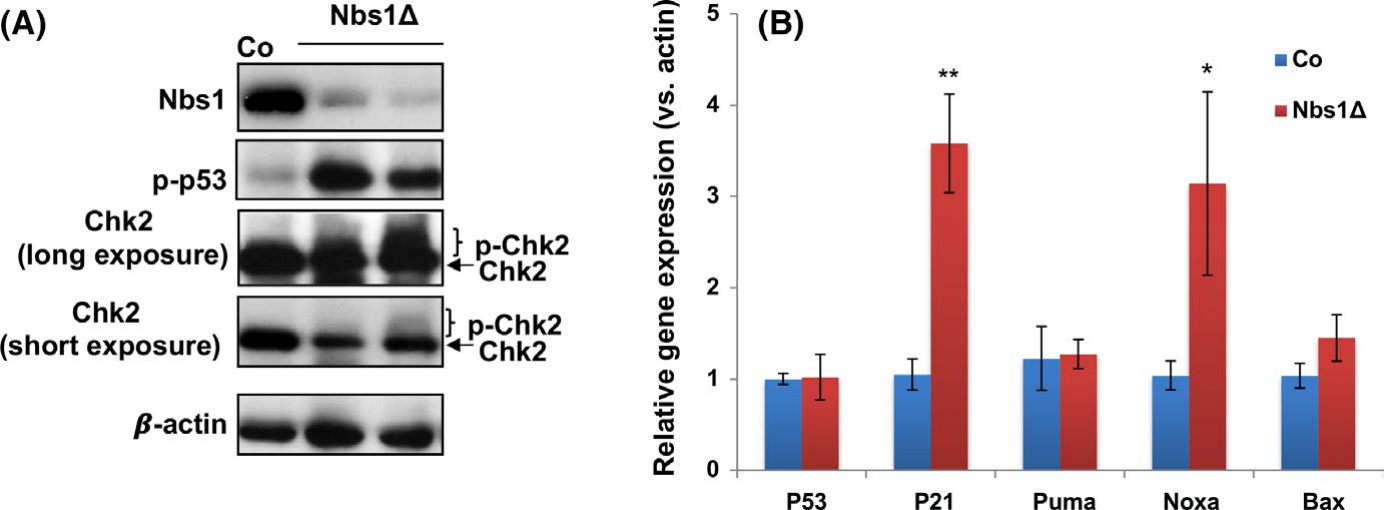
Activated DNA damage response in Nbs1‐HSCΔ foetal livers. A, Western blotting analysis on p53 and Chk2 phosphorylation in E15.5 control (Co) and Nbs1‐HSCΔ (Nbs1Δ) foetal liver samples. β‐Actin is used as the loading control. B, qRT‐PCR analysis of p53 and its downstream target genes (p21, Noxa, Puma and Bax) in E17.5 foetal livers from control (Co, N = 5) and Nbs1‐HSCΔ (Nbs1Δ, N = 3) mice embryos. The expression of individual gene in control samples was defined as ‘1’. β‐Actin expression level was used as the internal control. Note: *, *P* < .05; **, *P* < .01. Unpaired Student's *t* test is used

Nbs1 deficiency in somatic cells generates unrepaired DNA breaks and results in p53 stabilization and its transcriptional activity.^36^, ^39^ We found constitutive activation of the Atm‐Chk2‐p53 axis in Nbs1‐HSCΔ haematopoietic cells, as evidenced by enhanced phosphorylation of H2AX, Chk2 and p53 (Figure 5A) by Western blotting analysis and upregulation of p53 target genes, such as p21 and Noxa (Figure 5B) with qPCR assay. p53 signaling is one of the major cell fate determination pathways in HSCs.^12^, ^35^, ^48^ p53 loss has been proposed to be beneficial for the HSC survival after DNA damage induction by IR.^49^ Furthermore, p53 null could partially rescue the neural stem cell loss and mice lethality in the mouse model with Nbs1 specifically deleted in developing brain neocortex.^37^ To investigate whether the HSC defects and lethality of Nbs1‐HSCΔ mice are due to over‐activated p53 signaling, we genetically deleted p53 in Nbs1‐HSCΔ mice (Nbs1/p53‐ HSCΔ). To our surprise, p53 loss in Nbs1‐HSCΔ mice accelerates embryonic death (Figure S1G). We noticed that p53 deficiency accelerated the early onset of the anaemia phenotype as early as on E16 (Figure S3A). FACS analysis of Nbs1/p53‐HSC∆ foetal livers showed that p53 loss causes greater reduction of total cellularity and LK progenitors as compared with Nbs1‐HSCΔ foetal livers (Figure S3B‐C and data not shown). Thus, our data indicate that p53 activation upon the persistent DNA damages protects HSCs and LK progenitors from fast depletion.

### 3.6 p53 status determines cell fates of foetal HSCs and LK progenitors with persistent DNA breaks

**Figure 6:**
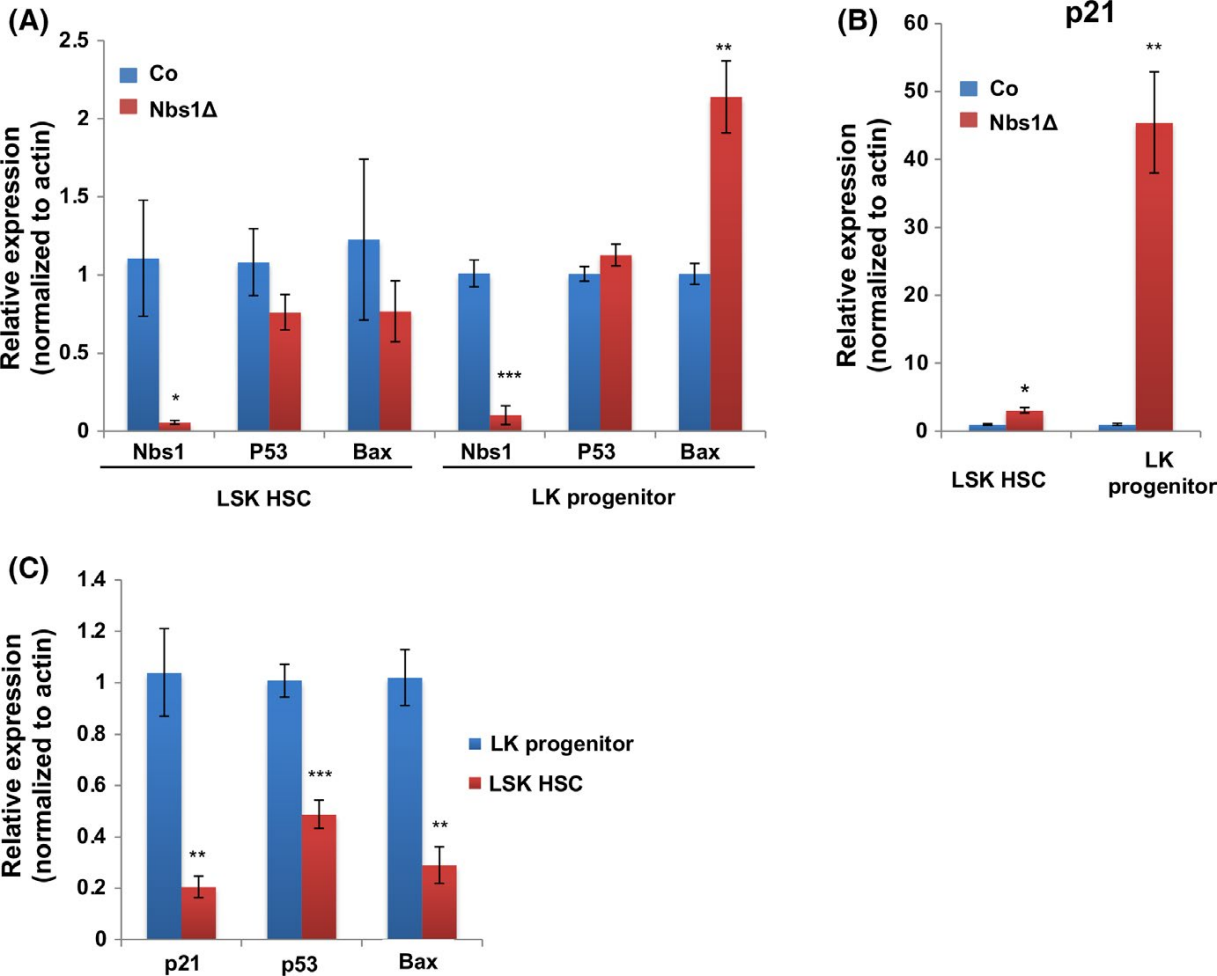
p53 signaling determines cell fates of Nbs1 deficient foetal liver HSC and LK progenitors. A, The expression of Nbs1, p53 and Bax in control (Co) and Nbs1‐HSCΔ (Nbs1Δ) HSCs and LK progenitors. The expression of these genes in control samples was defined as ‘1’. β‐Actin expression level was used as the internal control. N = 3 for each genotype. B, p21 expression in control (Co) and Nbs1 deficient (Nbs1Δ) HSCs and LK progenitors. The expression of p21 in control samples was defined as ‘1’. β‐Actin expression level was used as the internal control. N = 3 for each genotype. C, Relative expression of p21, p53, Bax in HSCs and LK progenitors from control mouse embryos. The expression of these genes in LK progenitors was defined as ‘1’. β ‐Actin expression level was used as the internal control. N = 3 for each group. Note: *, *P* < .05; **, *P* < .01; ***, *P* < .001. Unpaired Student's *t* test is used

To further investigate how HSCs and LK progenitors respond differentially to Nbs1 loss‐induced p53 signaling activation, we quantified the expression of p53 and its target genes in control and Nbs1 deficient HSCs and LK progenitors (E16.5). p53 mRNA levels did not change in HSCs and LK progenitors between control and Nbs1‐HSCΔ (Figure 6A), ruling out Nbs1’s effect at the transcriptional level but rather suggesting a post‐transcriptional regulation mediated by stabilized p53 upon DDR. qRT‐PCR analysis on Nbs1 deficient LK progenitors revealed greater induction of p53 target genes *p21* (45‐fold increases) and *Bax* (2‐fold increases) (Figure 6A‐B). In contrast, only mild induction of *p21* (3‐fold induction) was found in Nbs1 null HSCs (Figure 6B) and the pro‐apoptotic gene *Bax* was not upregulated (Figure 6A). These data further suggest that cell cycle arrest, for example mediated by p21, is predominantly executed in foetal liver HSCs, while the cell death programme, for example mediated by Bax, is a preferred choice for LK progenitors upon DNA damages (Figure 6A‐B).

Interestingly, in control mice, we noticed that apoptotic index in HSCs is less as compared with LK progenitors (Figure 4D). We measured the p53 mRNA levels in HSCs and LK progenitors in control mice. We found that foetal liver HSCs expressed lower level of p53 as compared with LK progenitors (Figure 6C). Thus, it is plausible that low level of p53 expression in HSCs could restrain the magnitude of p53 signaling activation and prevent HSC depletion when persistent DNA breaks exist.^8^, ^50^

## 4 DISCUSSION

DNA damage is one of the detrimental factors for HSC self‐renewal and differentiation.^8^, ^16^ Studies on quiescent HSCs in adult animals indicate that tissue and organismal ageing are partially mediated by accumulations of DNA damages inside stem cells.^51^, ^52^, ^53^ Defects in DNA damage repair machineries promote HSC ageing. The function of DNA repair during the HSC development remains largely unknown. In this study, using a mouse model with DNA double‐strand break repair molecule Nbs1 specifically deleted in foetal liver HSCs, we found that Nbs1‐mediated DSB repair machinery is essential for the foetal HSC development and haematopoiesis. Furthermore, foetal HSCs and haematopoietic progenitors respond differentially to persistent DNA breaks upon Nbs1 deficiency. In our study, we uncovered that the magnitude of p53 signaling in foetal HSCs and haematopoietic progenitors could be responsible for the distinct cell behaviour between these two cell populations.

### 4.1 Function of Nbs1‐mediated DDR in embryonic haematopoiesis

NBS1, together with MRE11 and RAD50, participates in DSB sensing, activation of ATM kinase. Furthermore, NBS1 could be phosphorylated by ATM and functions in fine‐tuning of DDR signaling and cell cycle regulation by affecting the phosphorylation of CHK2 and SMC1.^33^, ^42^ Recent findings show that NBS1 is essential for resolving the DNA replication fork stalling and replication intermediates.^36^ Previously, we and other groups found that Nbs1 deletions in different organs, such as developing brains, T‐cell progenitors, B‐cell progenitors and hair follicle (HF) progenitors, generate persistent DNA breaks and consequently result in developmental defects and defective tissue homeostasis.^37^, ^38^, ^39^, ^54^ By crossing the *Nbs1* ^f/f^ mouse with the Vav‐Cre transgenic mouse, we generated a mouse model with Nbs1 specifically deletion in developing HSCs (Nbs1‐HSCΔ mice). Nbs1‐HSCΔ mice are perinatally lethal due to defective haematopoiesis in embryonic foetal livers and newborn bone marrows, which is in accordance with recent findings from John Petrini's group.^55^ Our Nbs1‐HSCΔ mice show severe anaemia with great reduction in bone marrow cells as well as mature haematopoietic cells, which is mimicking the aplastic anaemia symptom with human NBS1 mutation.^40^ With in‐depth analysis on haematopoiesis during mouse development, we found genomes of Nbs1 deficient haematopoietic cells in foetal livers were enriched with chromosome abnormalities. Nbs1 deficient HSCs and haematopoietic progenitors showed high levels of DNA breaks, which activate Atm‐Chk2‐p53 signaling. p53 transcriptionally enhances expressions of p21 and Noxa, which cause cell cycle arrest and cell death of foetal liver haematopoietic cells, respectively.^5^, ^56^ Persistent DNA breaks and DDR activation upon Nbs1 deletion result in the clearance of haematopoietic progenitors due to hyper‐activation of p53 signaling. Intriguingly, although Nbs1 is essential for viability of proliferating cells, we noticed that the absolute number of HSCs is transiently increased in foetal livers from Nbs1‐HSCΔ mice. The phenomena could be due to the following facts. First, Nbs1 null HSCs are protected by cell cycle arrest but not depleted through apoptosis. Thus, HSC pool tends to be maintained. Furthermore, crisis of defective production of mature haematopoietic cells may trigger HSC dividing, and Nbs1 deficient HSCs could go through several rounds of cell divisions before they finally lose viability.

To summarize, our study discloses that persistent DNA damages in HSCs during embryonic development is detrimental to HSC dynamics and embryonic haematopoiesis. Via maintaining genomic stability in HSC and its progenies, Nbs1‐mediated DNA double‐strand break repair pathway safeguards haematopoiesis in embryonic and neonatal stages.

### 4.2 Cell fates of murine foetal liver HSCs and haematopoietic progenitors with persistent DNA lesions

Cell fates of HSCs and haematopoietic progenitors in mice and humans upon DNA damages are under debate. Previously, Mohrin *et al* showed that murine quiescent HSCs are resistant to cell death as compared to haematopoietic progenitors, which may be explained by cyto‐protective role of quiescence.^26^ Furthermore, in vivo, murine HSCs after medium dose of IR (2‐6Gy) tend to commit cellular senescence in order to maintain the HSC pool size.^57^ Intriguingly, active cycling human HSCs isolated from the cord blood are prone to p53‐mediated cell death upon IR.^27^ The discrepancy of the different HSC cell fate in human and mouse may be explained by the following possibilities: a) the species‐specific effect; b) cell cycle specific consequence; c) differential response of foetal HSCs vs. adult HSCs determined by their distinct transcriptional profiles.^12^, ^28^ In our study, we analysed cell fates of Nbs1 deficient HSCs and Lin^‐^Sca1^‐^c‐Kit^+^ haematopoietic progenitors in mouse foetal livers. We found that murine embryonic HSCs, similar to quiescent HSCs from adult mice, are resistant to DNA damages. HSCs with DSBs commit cell cycle arrest, while Lin^‐^Sca1^‐^c‐Kit^+^ haematopoietic progenitors, expressing higher level of p53 targets genes *p21* and *Bax*, show strong induction of apoptosis. Our finding suggests that cell cycle status,^57^ that is quiescence and cycling, is not a cell fate determinative factor for murine HSCs in response to persistent DNA damages. In this regard, the discrepancy on cell fate commitments of human cord blood‐derived HSCs and mouse HSCs in response to DNA breaks may be species‐specific.^12^, ^26^, ^27^, ^28^ Human and mouse HSCs are distinct on expressions of cell surface markers. Furthermore, they differ in heterogeneity of HSC pool, cell cycle status, even DNA repair dynamics.^58^ All these distinct properties might contribute to different cell fates of human and mouse HSCs upon DNA damages.

### 4.3 Role of p53 in cellular fitness of embryonic HSCs

p53 signaling determines stem cell behaviour upon stress condition. Deficiency of DNA repair machinery occasionally results in reduced cellular viability, partially through the activation of p53‐mediated cell death or cell cycle arrest. Thus, in central nervous system (CNS), loss of p53 could partially rescue the loss of proliferative neural stem cells and extend the lifespan of DNA repair deficient mice, as shown in Nbs1 CNS specific knockout mice, Brca1 CNS specific knockout mice,^59^ Ddb1 CNS specific knockout mice,^60^ TopBP1 CNS specific knockout mice,^61^ *etc.* Intriguingly, in other studies, p53 deletion exacerbates mice lethality as evidenced by studies on telomere dysfunction mice, Atr hypomorphic mice, Rad50 hypomorphic mice. For example, p53 loss in the third‐generation telomere dysfunction mice results in increases of intestinal stem cells with high degree of genomic instability. In *Nbs1* deficient hair follicle (HF) progenitors, p53 loss causes further upregulation of γ‐H2AX foci in the HF progenitors.^54^ These findings indicate that tissue‐specific stem cells could respond distinctively to DNA lesions generated by DNA repair deficiency. In our study, by crossing p53 null into Nbs1‐HSC∆ background, we found that p53 deficiency results in more profound loss of haematopoietic progenitors (LK progenitors) and mature haematopoietic cells, finally accelerating lethality of Nbs1‐HSC∆ mice. These phenomena are possibly caused by abnormal cell cycling and further DNA damage accumulation in Nbs1 null HSCs and haematopoietic progenitors since p53 null legitimates arrested LK progenitors/HSCs re‐entering into cell cycle. Furthermore, lower p53 expression is found in HSCs as compared with haematopoietic progenitors. In our study, we found persistent DNA damages upon Nbs1 deletion results in a low magnitude of p53 signaling in HSCs. ‘Low p53 expression’ in foetal liver HSCs plays a cyto‐protective role in maintaining the HSC pool size by preferably activating cell cycle arrest through mildly upregulating p21. Thus, our study indicates that proper p53 expression is vital for embryonic HSC development and haematopoiesis.

## CONFLICT OF INTEREST

The authors have declared no competing interests.

## AUTHOR CONTRIBUTIONS

Y. C. and T. L. performed most of the experiments, analysed and interpreted data. T. L. prepared the figures and the manuscript; J. S. and Z. J. helped in FACS analysis and sorting. T. L. and Z.‐Q. W. designed experiments, analysed data and composed the manuscript.

## Supporting information

Fig S1

Fig S2

Fig S3

## Data Availability

The data that support the findings of this study are available from the corresponding author upon request.
