# Supplementary figures and images for: Nbs1‐mediated DNA damage repair pathway regulates haematopoietic stem cell development and embryonic haematopoiesis
Source: Cell Prolif. 2021 Feb 14;54(3):e12972. doi: 10.1111/cpr.12972 (PMC7941224; doi:10.1111/cpr.12972)

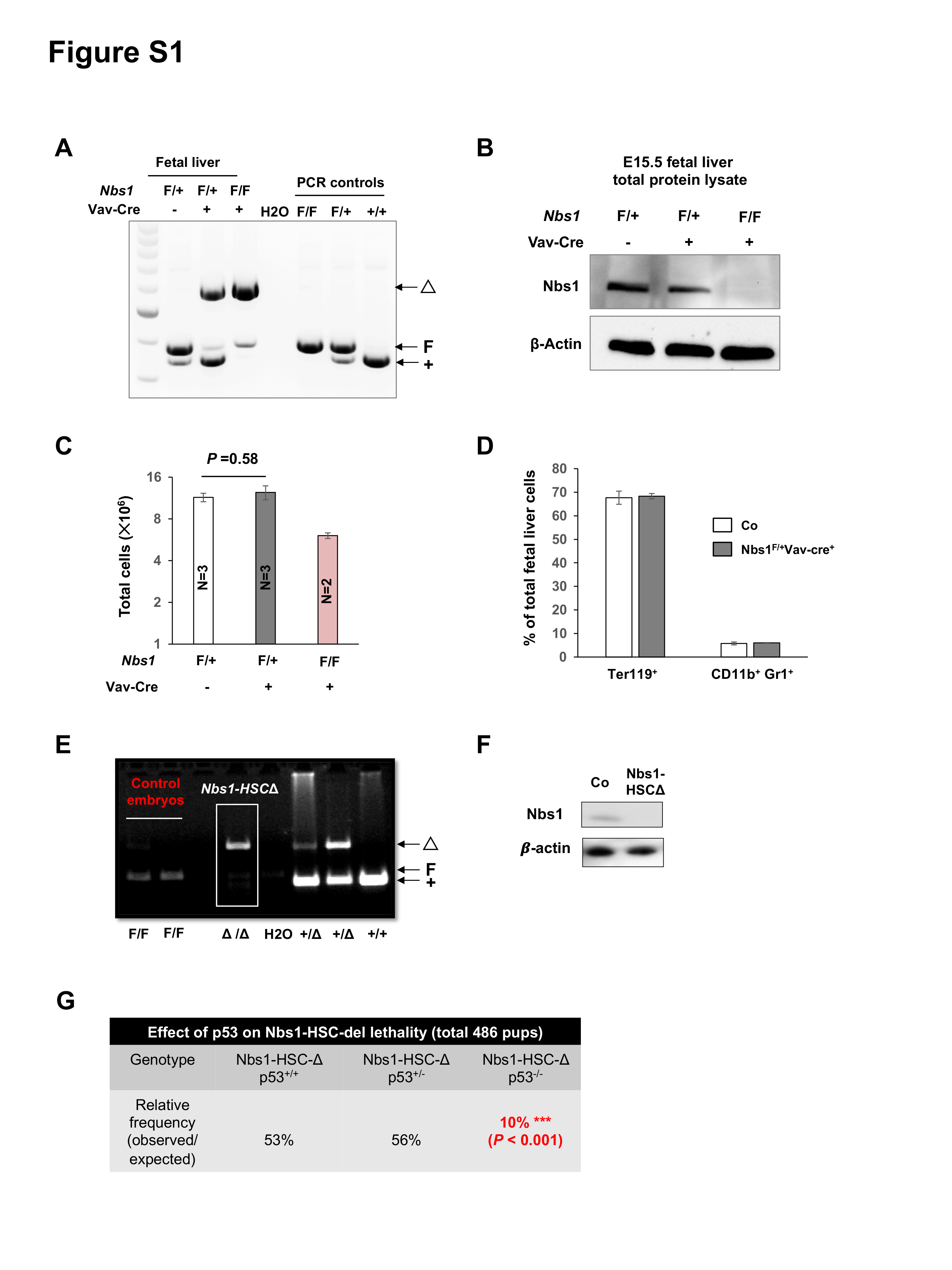

Supplement: Supplementary file 1 — Fig S1 [file CPR-54-e12972-s003.tif]

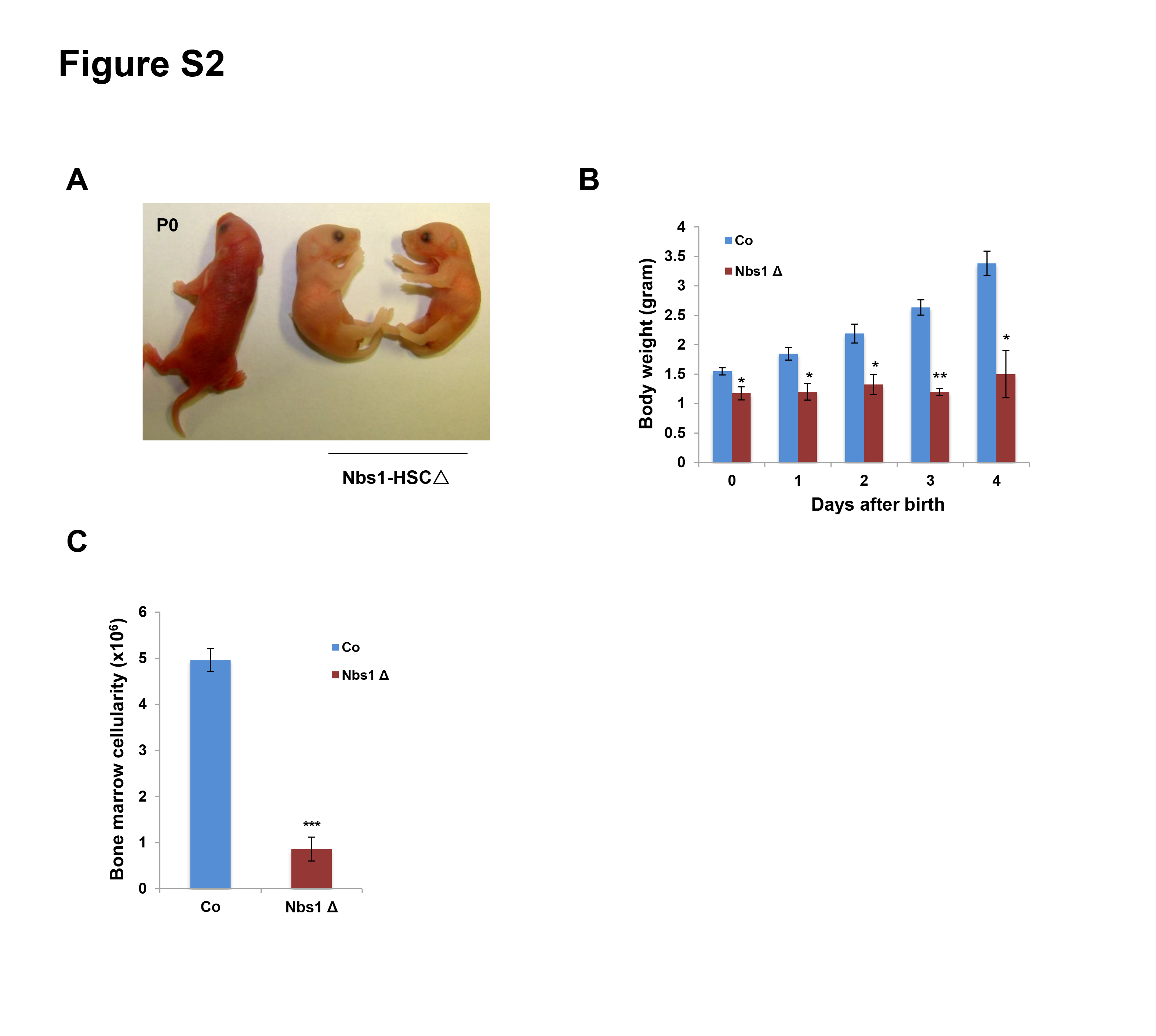

Supplement: Supplementary file 2 — Fig S2 [file CPR-54-e12972-s001.tif]

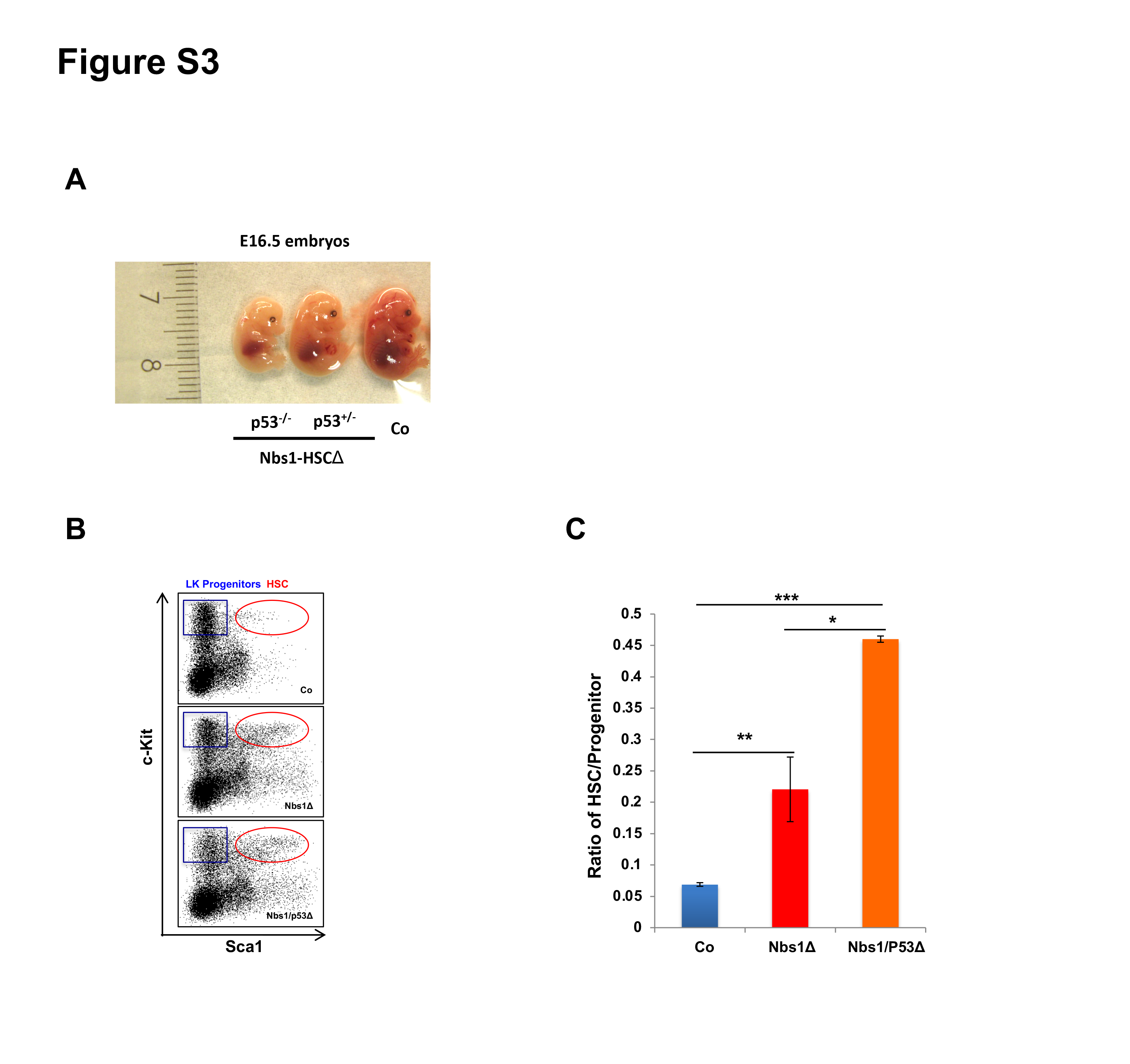

Supplement: Supplementary file 3 — Fig S3 [file CPR-54-e12972-s002.tif]
